# An Asp7Gly Substitution in PPARG Is Associated with Decreased Transcriptional Activation Activity

**DOI:** 10.1371/journal.pone.0086954

**Published:** 2014-01-23

**Authors:** Liushuai Hua, Jing Wang, Mingxun Li, Xiaomei Sun, Liangzhi Zhang, Chuzhao Lei, Xianyong Lan, Xingtang Fang, Xin Zhao, Hong Chen

**Affiliations:** 1 College of Animal Science and Technology, Northwest A&F University, Shaanxi Key Laboratory of Molecular Biology for Agriculture, Yangling, Shaanxi, China; 2 Guangdong Provincial Public Laboratory for Wild Animal Conservation and Management, Guangdong Entomological Institute (South China Institute of Endangered Animals), Guangzhou, China; 3 Institute of Cellular and Molecular Biology, Jiangsu Normal University, Xuzhou, Jiangsu, China; INRA, France

## Abstract

As the master regulator of adipogenesis, peroxisome proliferator-activated receptor gamma (PPARG) is required for the accumulation of adipose tissue and hence contributes to obesity. A previous study showed that the substitution of +20A>G in *PPARG* changed the 7^th^ amino acid from Asp to Gly, creating a mutant referred to as PPARG Asp7Gly. In this study, association analysis indicated that PPARG Asp7Gly was associated with lower body height, body weight and heart girth in cattle (*P*<0.05). Overexpression of PPARG in NIH3T3-L1 cells showed that the Asp7Gly substitution may cause a decrease in its adipogenic ability and the mRNA levels of CIDEC (cell death-inducing DFFA-like effector c) and aP2, which are all transcriptionally activated by PPARG during adipocyte differentiation. A dual-luciferase reporter assay was used to analyze the promoter activity of CIDEC. The results confirmed that the mutant PPARG exhibited weaker transcriptional activation activity than the wild type (*P*<0.05). These findings likely explain the associations between the Asp7Gly substitution and the body measurements. Additionally, the Asp7Gly mutation may be used in molecular marker assisted selection (MAS) of cattle breeding in the future.

## Introduction

Adipose tissue is essential for whole body energy homeostasis. This tissue serves as a safe place for storing excess energy to avoid lipid buildup in other tissues, and it releases energy when other tissues are in need [1]. The peroxisome proliferator-activated receptors (PPARs) are a group of nuclear receptor proteins that play essential roles in the regulation of energy homeostasis, adipogenesis and lipid metabolism. Three types of PPARs have been identified: alpha, delta and gamma (PPARA, PPARD and PPARG). As the master regulator of fat cell formation, peroxisome proliferator-activated receptor gamma (PPARG) is required for the accumulation of adipose tissue and hence contributes to obesity [2], [3]. The functional importance of PPARG makes its mutations potentially have great impacts on adipose accumulation. A number of *PPARG* genetic variants have been identified [4]. For example, the highly prevalent Pro12Ala substitution in human PPARG was associated with a lower body mass index (BMI), improved insulin sensitivity, decreased insulin resistance and a decreased risk of type 2 diabetes [5]–[7]. In our previous study, three mutations under linkage disequilibrium were detected in the *PPARG* gene that were associated with lower body measurements in cattle [8]. In these three mutations, a substitution of +20A>G changed the 7^th^ amino acid of PPARG from Asp to Gly, creating a mutant referred to as PPARG Asp7Gly. Sequence analysis showed that Asp7Gly in bovine PPARG and Pro12Ala in human PPARG were located within the same structural domain, which suggested that an understanding of the biological effects of Asp7Gly could help to explain the variability in cattle body measurements. The associations between Asp7Gly and body measurements were verified in a new herd, and the two *PPARG* haplotypes (PPARG Asp7 and PPARG Gly7) were overexpressed in NIH3T3-L1 cells (a pre-adipocyte cell line) to determine the effects of these mutations on adipogenesis. This study may enhance our understanding of PPARG regulation and help animal scientists to develop genetic markers.

## Methods and Procedures

### Ethics statement

This study was approved by the ethics committee of the Bureau of Animal Husbandry in Pingdingshan (Henan, China), and all efforts were made to minimize suffering.

### Animals and management

Blood samples were obtained from 121 individual Jiaxian cattle, which is a famous indigenous cattle breed in China. The cattle were reared under the same nutrition conditions as those used at the Henan Jiaxian Cattle Breeding Center (Pingdingshan, China). Body measurements, including body height, height at hip cross, body length, hip width, heart girth, rump length, hucklebone width and body weight of individuals older than 2 years old, were collected at the same time.

### PPARG Asp7Gly genotyping

The genotyping of PPARG Asp7Gly followed the method used in previous work [8]. First, DNA samples were extracted from blood samples. Subsequently, forced PCR-RFLP was used to investigate the genotype of each individual. The primers used for this amplification were as follows: forward, 5′ AAACGGACGTCTTGGCTCATT 3′; reverse, 5′ CTCTCTGGGTCAATAAGAGGA 3′. The mutant individuals (PPARG Gly7) could be detected using the restriction endonuclease *Sau*96I.

### RNA isolation, cDNA synthesis and plasmids constructions

Cattle adipose tissue was collected from a slaughterhouse owned by Shaanxi Kingbull Livestock Co., Ltd. (Shaanxi China), and we obtained permission to use these animal parts from the company prior to sampling. The tissue samples were immediately frozen in liquid nitrogen prior to storage at −80°C in the lab. Total RNA was isolated from the frozen adipose tissue using Trizol reagent (TaKaRa, Dalian, China). The quantity and quality of the RNA were determined using a NanoPhotometer spectrometer (Implen Inc., CA, USA). Total RNA was reverse-transcribed with the PrimeScript reverse transcription polymerase chain reaction (RT-PCR) Kit (TaKaRa).

Constructions of pEGFP-C1-PPARG-WT/MT: The primers for the wild type (WT) and mutant (MT) PPARG are shown in Table 1. The coding DNA sequence (CDS) regions of WT and MT PPARG were PCR amplified from the cDNA sample. The length of the CDS region was 1518 bp, which encoded 505 amino acids. After double digestion with *Eco*RI and *Kpn*I, ligation with T4 ligase and screening in competent *Escherichia coli* Top10 cells (Tiangen, Beijing, China), the vectors pEGFP-C1-PPARG-WT and pEGFP-C1-PPARG-MT were validated by double digestion and sequencing. The WT and MT PPARG were identical except for the 7^th^ amino acid, which was changed from Asp to Gly.

**Table 1:** The primers used to amplify wild type and mutant *PPARG* alleles.

Primer names	Primer sequences
PPARG-WT-F	CCGGAATTCG**ATG**GGTGAAACTCTGGGAGATGCTC
PPARG-MT-F	CCGGAATTCG**ATG**GGTGAAACTCTGGGAGGTGCTC
PPARG-R	CGGGGTACC **CTA**ATACAAGTCCTTGTAGATTTCC

Notes: The underlined sequences indicate the enzyme cutting sites *Eco*RI (GAATTC) and *Kpn*I (GGTACC), which were artificially added into the forward and reverse primers, respectively. The sequences in bold show the translation start site (ATG) and translation stop site (CTA, which is the reverse complement of the TAG). The sequences in frame show the Asp7Gly mutation (the codon was changed from GAT to GGT).

Constructions of pGL3-CIDEC-promoter: The promoter region of cell death-inducing DFFA-like effector c (CIDEC) was PCR amplified from cattle genomic DNA. The primers for the *CIDEC* promoter region were as follows: sense, 5′- GTAGAATTGCTGGGTCACGGAT -3′; antisense, 5′- CCTGGAGAGGGACTTAGGGTAG -3′, and the length of this DNA fragment was 2069 bp. The pGL3-CIDEC-promoter was constructed by inserting the promoter fragment into pGL3-basic vector.

Constructions of pcDNA-PPARG-WT/MT: The WT and MT PPARG were sub-cloned into the pcDNA3.1 vector to construct pcDNA-PPARG-WT and pcDNA-PPARG-MT.

### Cell culture, transfection and lipid staining

In order to determine their inducing adipogenesis ability, the two forms of PPARG (WT and MT) were overexpressed in the NIH3T3-L1 cells. NIH3T3-L1 cells (purchased from cellbank.org.cn) were cultured in a growth medium consisting of Dulbecco's Modified Eagle's Medium (DMEM) (Hyclone, Logan, UT) with 10% fetal bovine serum (FBS) (HyClone) and 1× antibiotics (100 U/ml penicillin, 100 µg/ml streptomycin; KeyGEN, Nanjing, China) and incubated at 37°C with 5% CO_2_.

pEGFP-C1-PPARG-WT and pEGFP-C1-PPARG-MT were transfected into confluent NIH3T3-L1 cells. The cells were divided into two groups with three replicates: the growth group was exposed to growth medium (the NIH3T3-L1 cells will keep undifferentiated in growth medium), and the differentiation group was exposed to DEX medium (growth medium containing 1 µmol/L DEX (Promega, Heidelberg, Germany)) (Dex medium has a weak ability to induce the NIH3T3-L1 cells differentiate into adipocytes). pEGFP-C1-basic was transfected into NIH3T3-L1 cells under the same conditions (two groups with three replicates) as a negative control. Lipid accumulation in adipocytes was used to indicate the adipose differentiation status.

Transient transfections were performed with Lipofectamine 2000 transfection reagent (Invitrogen, Carlsbad, CA). First, DNA and Lipofectamine 2000 were diluted in DMEM medium separately at a ratio of 1∶2.5. After incubation for 5 minutes at room temperature, the diluted DNA and Lipofectamine 2000 were mixed together and incubated for 20 minutes at room temperature. This complex was then added to a culture dish containing cells and growth medium. The cells were incubated at 37°C with CO_2_ in an incubator for 24 hours before assaying transgene expression.

Lipid accumulation in adipocytes was visualized by staining with Oil Red O (ORO) [9]. First, the cells were rinsed twice with phosphate buffered saline (PBS) and fixed with 10% formaldehyde for 1 h at room temperature. After 2 washes with PBS, the cells were stained for at least 20 min in freshly diluted ORO solution (0.3% (w/v) ORO in 60% isopropanol. The staining solution was then removed, and the cells were washed twice with PBS. ORO was extracted with isopropanol and quantified spectrophotometrically (NanoPhotometer, Implen Inc., CA, USA) at 530 nm [9], [10].

### Q-PCR

To verify similar transfection efficiencies between groups, quantitative PCR (Q-PCR) was used to detect the relative expression levels of the two PPARG haplotypes. The sense primer was 5′-TGCAAGGACCTCACAAGA-3′, and the antisense primer was 5′- ATGCTGGAGAAGTCAACG -3′. D-glyceraldehyde-3-phosphate dehydrogenase (GAPDH) was used as an internal reference. The sense primer for GAPDH was 5′-AACTTTGGCATTGTGGAAGG-3′, and the antisense primer was 5′-CCCTGTTGCTGTAGCCGTAT-3′ [11]. The Q-PCR was performed with a SYBR Green PCR kit (TaKaRa) using 20 µl of reaction solution containing 10 µl of 2×SYBR Green PCR master mix, 20 ng of cDNA and primers (200 nM). Each reaction was carried out in triplicate and run in a CFX96 real-time PCR detection system (Bio-Rad, Hercules, CA, USA). If there were no significant differences between the expression levels of PPARG, we assumed that the transfection efficiencies were identical between the groups.

To detect the transcriptional activation activity changes of PPARG, the expression pattern of CIDEC and aP2 were determined by Q-PCR. After transfection, the pEGFP-C1-PPARG-WT, pEGFP-C1-PPARG-MT and pEGFP-C1-basic groups were fed with growth medium and harvested every 24 hours, and then the RNA was isolated and cDNA was synthetized. The specific primers for *CIDEC* gene were as follows: sense, 5′- AGCTAGCCCTTTCCCAGAAG -3′; antisense, 5′- CCTTGTAGCAGTGCAGGTCA -3′ [12]. The primers for *aP2* gene were: sense, 5′- GCGTGGAATTCGATGAAATCA -3′; antisense, 5′- CCCGCCATCTAGGGTTATGA -3′[13].

### Dual-luciferase reporter assay

To verify differences in transcriptional activation activity between the WT and MT PPARG, the promoter activity of CIDEC was determined using the dual-luciferase reporter assay kit (Promega). NIH3T3-L1 cells were seeded onto 24-well plates at the required density. After 18 h, the cells were co-transfected with the luciferase reporter construct pGL3-CIDEC-promoter (firefly fluorescence reporter) and pRL-TK plasmid (renilla fluorescence reporter) at a ratio of 25∶1. Seventy-two hours post-transfection, the cells were passively lysed for the promoter assay. The luciferase activity was quantified according to the manufacturer's instructions. Cells co-transfected with pGL3-basic (firefly fluorescence) + pRL-TK (renilla fluorescence) were used as a negative control, and pGL3-control + pRL-TK were co-transfected as a positive control. The promoter activity of the target DNA fragment was equal to the ratio of firefly and renilla fluorescence intensity corrected using empty cells and the control group. The promoter activity detection was repeated three times in parallel for statistical analysis.

### Statistical analysis

The general linear model (GLM) in SPSS (version 13.0) was used to evaluate the associations between the PPARG genotypes and the body measurements of cattle. The independent variables were PPARG genotype, age and sex. The GLM was displayed as follows: Y*_ijk_*  =  μ + G*_i_* + A*_j_* + S*_k_* + e*_ijk_*, where Y*_ijk_*  =  observed value; μ  =  overall mean for each trait; G*_i_*  =  fixed effect associated with the i_th_ genotype; A*_j_*  =  fixed effect associated with the j_th_ age; S*_k_*  =  fixed effect associated with the k_th_ sex; and e*_ijk_*  =  random error.

A one-way analysis of variance (ANOVA) was used to compare the differences between the WT and MT PPARG groups. The results of the multiple comparisons were corrected by Bonferroni correction, and the differences were considered significant if *P*<0.05.

## Results

### Asp7Gly was associated with lower body measurements in Jiaxian cattle

Genomic DNA was successfully extracted from 121 blood samples of Jiaxian cattle, and PPARG Asp7Gly was genotyped in this population using forced-PCR-RFLP. Two genotypes, AA (n = 109) and AG (n = 12), were detected in the 121 individuals. The allele frequencies of A and G were 0.95 and 0.05, respectively. Considering genotype, gender and age factors, the associations between Asp7Gly and the body measurements of Jiaxian cattle were analyzed using the GLM model. The results verified that individuals with the Asp7Gly mutation had lower body height (*P*<0.05), body weight (*P*<0.05) and heart girth (*P*<0.01) (Table 2).

**Table 2:** Associations between PPARG Asp7Gly and body measurements of Jiaxian cattle.

Body measurement	Genotype	N	Mean	Standard deviation	Test of equality of variances	*P* value
Body height	AA	109	125.511	5.035	0.785	0.027
	AG	12	122.125	4.339		
Height at hip cross	AA	109	125.145	6.955	0.497	0.074
	AG	12	121.417	5.013		
Body length	AA	109	151.484	10.024	0.507	0.235
	AG	12	147.833	10.382		
Hip width	AA	109	44.762	3.546	0.058	0.467
	AG	12	44	1.954		
Heart girth	AA	109	178.311	8.113	0.494	0.009
	AG	12	171.75	8.072		
Rump length	AA	109	48.491	3.551	0.862	0.826
	AG	12	48.25	4.026		
Hucklebone width	AA	109	24.615	4.327	0.873	0.391
	AG	12	25.75	4.393		
Body weight	AA	109	483.434	61.903	0.801	0.02
	AG	12	438.813	67.051		

### Asp7Gly damaged the adipogenic ability of PPARG

**Figure 1:**
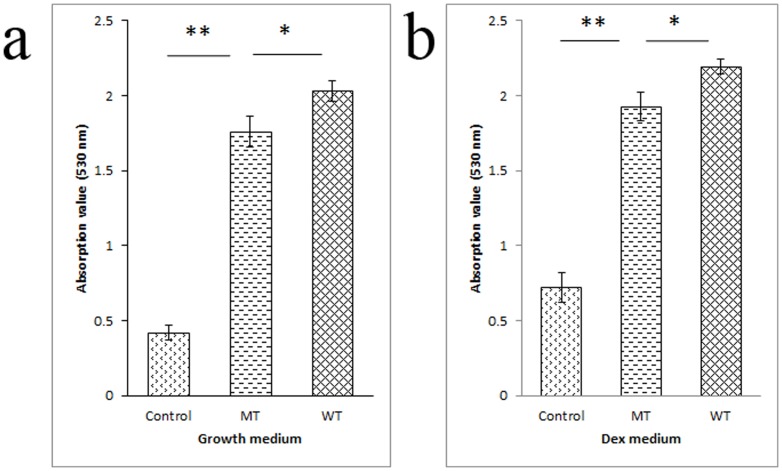
Quantification of the adipogenic ability of PPARG. Overexpression of PPARG induced the differentiation of NIH3T3-L1 cells under either growth conditions (growth medium) or differentiation conditions (DEX medium), while the mutant (MT) PPARG had lower adipogenic ability compared with the wild type (WT). The WT and MT groups were transfected with pEGFP-C1-PPARG-WT and pEGFP-C1-PPARG-MT vectors, respectively, and the control group was transfected with the pEGFP-C1-basic vector. The differentiation status was quantified spectrophotometrically based on the quantity of ORO extracted from NIH3T3-L1 cells, and three repetitions were performed for each treatment. The results confirmed that the mutant PPARG had lower adipogenic ability under both growth conditions (a) and differentiation conditions (b). The symbols “*”, “**” and “***” in figure indicate the *P*<0.05, *P*<0.01 and *P*<0.001, respectively.

Because overexpression of PPARG can induce the differentiation of pre-adipocytes, the adipose differentiation status indicates the adipogenic ability of PPARG. After transfection, green fluorescence was detected in the pEGFP-C1-PPARG-WT, pEGFP-C1-PPARG-MT and pEGFP-C1-basic groups, confirming successful transfection in all groups. Q-PCR showed that there were no differences in the relative expression levels of PPARG between the WT and MT groups (*P*>0.05), suggesting similar transfection efficiencies (Table 3). After staining, the ORO in cells was extracted and quantified spectrophotometrically. The results proved that the mutant PPARG was less capable of inducing adipogenesis under both growth conditions (Figure 1a) and differentiation conditions (Figure 1b) (*P*<0.05).

**Table 3:** Expression levels of the two PPARG forms.

Groups	Replicates	Mean	SD	*P* value
WT	3	15074.333	1065.501	0.926
MT	3	14969.667	1493.527	

### Asp7Gly decreased the PPARG-induced transcription of CIDEC and aP2

**Figure 2:**
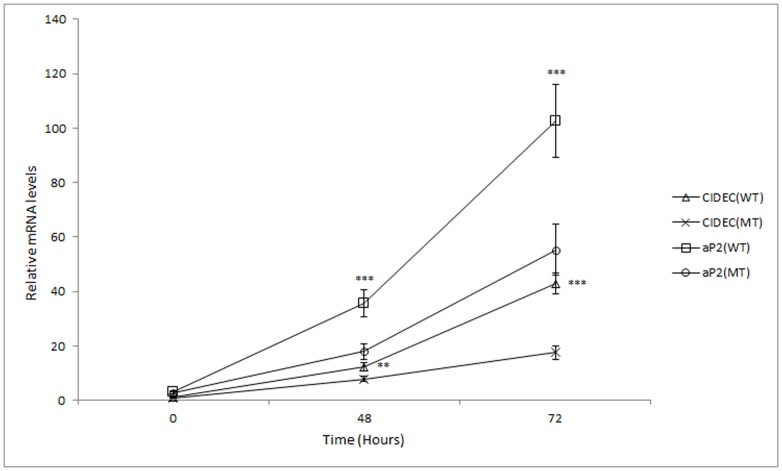
Quantification of the CIDEC and aP2 expression pattern. Q-PCR was used to determine the expression pattern of CIDEC and aP2. The figure shows the relative expression of CIDEC and aP2 in WT and MT groups (3T3-L1 cells transfected with pEGFP-C1-PPARG-WT and pEGFP-C1-PPARG-MT vectors, respectively). The gene expression in the control group (3T3-L1 cells transfected with pEGFP-C1-basic vector) was used as the reference. Overexpression of PPARG induced the transcription of CIDEC and aP2; however, the mRNA levels of CIDEC and aP2 were both lower in the MT group 48 hours after transfections (*P*<0.05).

PPARG is a ligand-dependent transcription factor, which binds to the PPREs (PPAR response elements) of target genes to regulate their expression. PPREs are located in the promoter region of a gene, and when PPARG binds its ligand, the transcription of target genes is either increased or decreased depending on the gene. It is demonstrated that the *CIDEC* and *aP2* genes contain PPREs and has been shown to be directly regulated by PPARG [14], [15]. The expression pattern of CIDEC and aP2 during adipogenesis could be used to indicate the transcriptional activation activity of PPARG. Q-PCR results showed that overexpression of PPARG induced the transcriptions of CIDEC and aP2 in NIH3T3-L1 cells, while the mRNA levels were lower in the pEGFP-C1-PPARG-MT group compared with that in the pEGFP-C1-PPARG-WT group 48 hours after transfection (*P*<0.05) (Figure 2).

### Asp7Gly decreased the promoter activity of CIDEC

**Figure 3:**
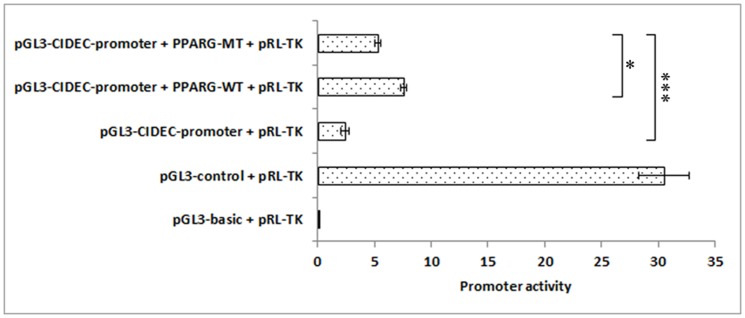
Analysis of *CIDEC* promoter activity driven by PPARG. A dual-luciferase reporter assay was used to detect the promoter activity of CIDEC driven by PPARG. The results obtained with the pGL3-basic and pGL3-control groups indicated that the dual-luciferase reporter assay was functional. The *CIDEC* promoter processes weak promoter activity in NIH3T3-L1 cells, and its activity was increased by the overexpression of PPARG (*P*<0.01). Although both wild type and mutant PPARG are able to initiate the expression of CIDEC, the Asp7Gly mutation decreased the transcriptional activation activity of PPARG significantly (*P*<0.05).

Dual-luciferase reporter assay confirmed that the 2069-bp DNA fragment upstream *CIDEC* gene exhibits promoter activity and could be activated by PPARG (*P*<0.01); however, MT PPARG was a weaker transcriptional activator compared with WT (*P*<0.05) (Figure 3).

## Discussion

In this study, the Asp7Gly mutation was found to be associated with decreased body measurements in cattle. A possible explanation is that this mutation decreases the adipogenic ability of PPARG. Overexpression of the two types of PPARG proved that the mutant PPARG had weaker adipogenic ability, but the underlying mechanism is worth further investigations. There are three isotypes of PPARs, although they share significant structural similarity, the biological effects associated with each PPAR isotype are distinct. For example, PPARA and PPARD regulate fatty acid catabolism, whereas PPARG controls lipid storage and adipogenesis. The different functions of PPARs in vivo can be explained in part by the different structures of their N termini [16]. This suggests that the N terminus of the PPARs is closely linked with its specific functions. There are two isoforms of the *PPARG* gene as a result of alternative mRNA splicing. The PPARG1 isoform is expressed in most tissues, whereas PPARG2 is specific for adipose tissue, where it plays a key role in regulating adipogenic differentiation [17]. PPARG2 has an additional 28 amino acids at its amino terminus that render its ligand-independent activation domain 5-10-fold more effective than that of PPARG1. The functional differences between the two PPARG isoforms also imply that the N terminus of PPARG is pivotal to its function. Additionally, the validated function of Pro12Ala in human PPARG also supports this hypothesis. The Pro12Ala mutation in human PPARG, which was the result of a CCA-to-GCA missense mutation in the NH2-terminal residue, showed decreased binding affinity for the cognate promoter element and reduced ability to transactivate responsive promoters [5], [18]. In this study, the Asp7Gly also located at the NH2-terminus of PPARG in cattle, suggesting that the Asp7Gly may cause a decrease in the transcriptional activation activity. CIDEC and aP2 are important for the regulation in lipid accumulation in adipocytes, and both contain a PPRE in their promoter region, indicating that they could be directly activated by PPARG [19]. The expression of CIDEC and aP2 were induced by PPARG overexpression, while the mRNA levels were lower in the MT group compared with the WT group, proving that Asp7Gly decreases the transcriptional activation activity of PPARG. The analysis of *CIDEC* promoter activity via the dual-luciferase reporter assay further confirmed this inference. Overexpression of both PPARG alleles could improve the promoter activity significantly; however, the mutant allele demonstrated weaker transcriptional activation compared with the wild type allele. Although we have proven that Asp7Gly decreased the transcriptional activation activity of PPARG and damaged its ability to induce adipogenesis, more work is needed to understand its specific mechanism; for example, the effect of the mutation on the conformation of PPARG and the influence of the mutation on the binding affinity of PPARG should be explored. It is worth to mention that measuring the traits related to obesity or meat quality (such as, marbling, fat content, fat color and tenderness) directly is more efficient for further association studies, but getting these data in large populations is hard and expensive. An alternative choice is to determine the body measurements or growth traits, since the body measurements always closely related to obesity status or fat deposition. For example, the BMI (body mass index) is usually used to indicate the obesity status[5].

The increased amount of molecular markers associated with cattle performance were reported [20], [21], but few biological mechanisms underlying these molecular markers had been explored. In this study, we proved that the Asp7Gly decreased the transcriptional activation activity of PPARG and damaged its adipogenic ability. These findings likely explain the associations between the Asp7Gly substitution and the body measurements, suggesting the Asp7Gly may be used in molecular marker assisted selection of cattle breeding in the future.
